# Supplementary material for: FAK activity exacerbates disturbed flow-mediated atherosclerosis via VEGFR2-CBL-NF-κB signaling
Source: J Biol Chem. 2025 Jun 14;301(7):110383. doi: 10.1016/j.jbc.2025.110383 (PMC12274819; doi:10.1016/j.jbc.2025.110383)
Supplement: Supplementary Material [file mmc1.docx]

**FAK activity exacerbates disturbed flow-mediated atherosclerosis**

**via VEGFR2-CBL-NF-κB signaling.**

James M. Murphy^a,#^, Duyen Thi Kieu Tran^a,#^, Kyuho Jeong^b,c,#^, Ly Nguyen^a^, Mai Thi Nguyen^a,^, Dhananjay Tambe^d^, Hanjoong Jo^e^, Eun-Young Erin Ahn^f^ and Ssang-Taek Steve Lim^a,*^

^#^Equally contributed

^a^Department of Pathology, University of Alabama at Birmingham, Birmingham, AL 35294

^b^Department of Biochemistry and Molecular Biology, University of South Alabama College of Medicine, Mobile, AL 36688

^c^Current address: Department of Biochemistry, College of Medicine, Dongguk University, Gyeongju, 38066 Korea.

^d^Department of Bioengineering, University of South Alabama College of Medicine, Mobile, AL 36617

^e^Department of Bioengineering, Emory University and Georgia Institute of Technology, Atlanta, GA 30322

^f^Department of Pathology, O’Neal Comprehensive Cancer Center, University of Alabama at Birmingham, Birmingham, AL 35294

Running title: Flow-mediated NF-κB signaling by FAK-VEGF2-Cbl axis

***Correspondence to:** Steve Lim, Ph.D., Department of Pathology, Division of Molecular Cellular Pathology, University of Alabama at Birmingham, 1825 University Blvd, Shelby 815, Birmingham, AL 35294

Tel.: (205) 975-4984; E-mail: stevelim@uabmc.edu

Key words: FAK, NF-κB, CBL, VEGFR2, disturbed flow, Atherosclerosis

Word Count: 4745

Number of figures: 6

**Supplemental Information**

**Supplemental methods**

**Flow Experiment**

HUVECs were transfected with either HA-CBL-WT or HA-CBL-Y3F overnight. HUVECs were then subjected to oscillatory flow for either 0 or 30 min. Cells were lysed in SDS loading buffer and subjected to immunoblotting.

**Supplemental Figure legends**

**Supplemental Figure1.** HUVECs were starved for 12 h in 0.2% FBS DMEM with or without VEGF (5 ng/ml) prior to initiation of **(A)** laminar flow (12 dynes/cm^2^) or **(B)** disturbed flow (5 dynes/cm^2^). **(A)** Representative immunoblotting of active FAK (pY397), active NF-κB p65 (pS536 p65), active IKKα/β (pS176/177), IκBα, and β-actin as loading control (n=3). **(B)** Representative immunoblotting of VCAM-1, active FAK (pY397 FAK), total FAK active NF-κB p65 (pS536 p65) and β-actin as loading control (n=3).

**Supplemental Figure 2.** HUVECs were transfected with either HA-CBL-WT or HA-CBL-Y3F overnight. HUVECs were then subjected to oscillatory flow for either 0 or 30 min. Representative immunoblotting for VEGFR2, active pY397 FAK, HA (CBL), or GAPDH as loading control (n=3).

**Supplemental Figure 3.** HUVECs were starved for 12 h in 0.2% FBS DMEM with or without FAK-I (2.5 uM) prior to initiation of disturbed flow (5 dynes/cm^2^). Immunostaining for EEA1 (green), VEGFR2 (red) and DNA (DAPI, blue). Scale bar: 20 μm.

**Supplemental Figure 4.** Partial carotid ligation (PCL) was performed on *Apoe-/-* mice and were treated with vehicle or FAK inhibitor (FAK-I, 30 mg/kg, twice daily) while on a western diet (WD) for 2 weeks. **(A)** Immunoblotting of lung lysates for FAK, active pY397 FAK, and either GAPDH or β-actin for loading controls. Immunostaining of carotid arteries for **(B)** pS536 NF-kB (Red) or **(C)** VCAM-1 (Red), or **(D)** CD68 (Green). **(B and C)** Endothelial cells were stained with vWF (Green). Nuclei with DAPI (Blue). L: Lumen, LC: Lipid Core, M: Media. Scale bar: 20 μm.

**Supplemental Figure 5.** Partial carotid ligation (PCL) was performed on *Apoe-/-;FAK-WT* and *Apoe-/-;FAK-KD* mice, and fed a western diet (WD) for 2 weeks. Immunostaining of carotid arteries for **(A)** VCAM-1 (Red) or **(B)** pS536 NF-kB (Red). Endothelial cells were stained with vWF (Green), and nuclei were stained with DAPI (blue). Scale bar: 20 μm.
