# Supplementary figures and images for: FAK activity exacerbates disturbed flow-mediated atherosclerosis via VEGFR2-CBL-NF-κB signaling
Source: J Biol Chem. 2025 Jun 14;301(7):110383. doi: 10.1016/j.jbc.2025.110383 (PMC12274819; doi:10.1016/j.jbc.2025.110383)

**A**

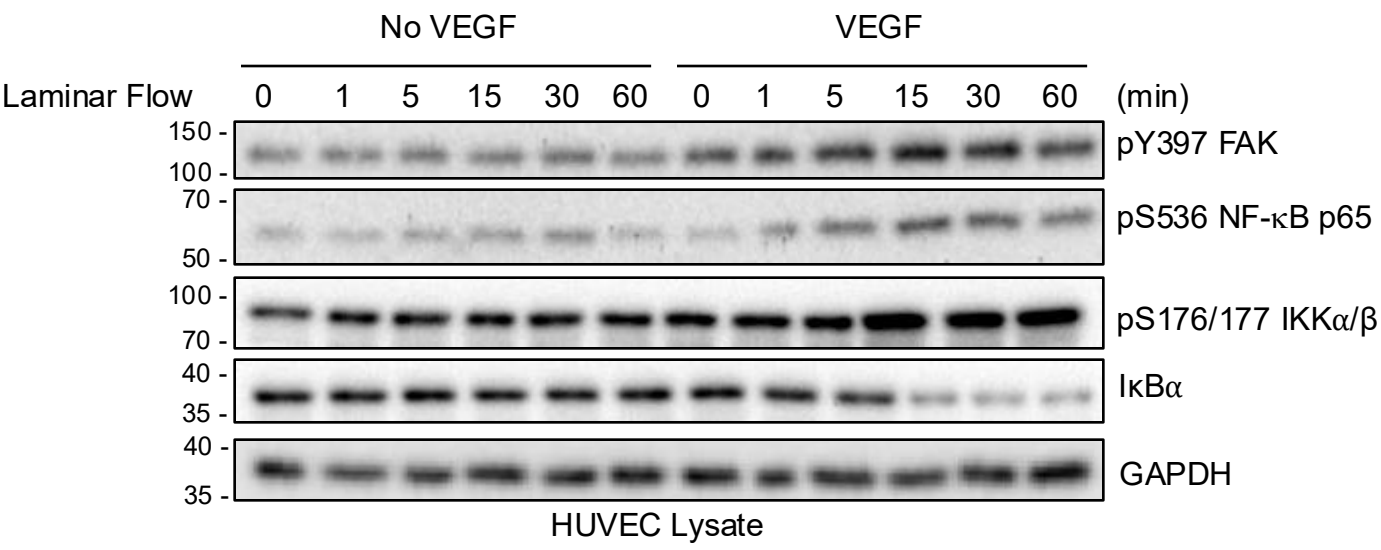

**B**

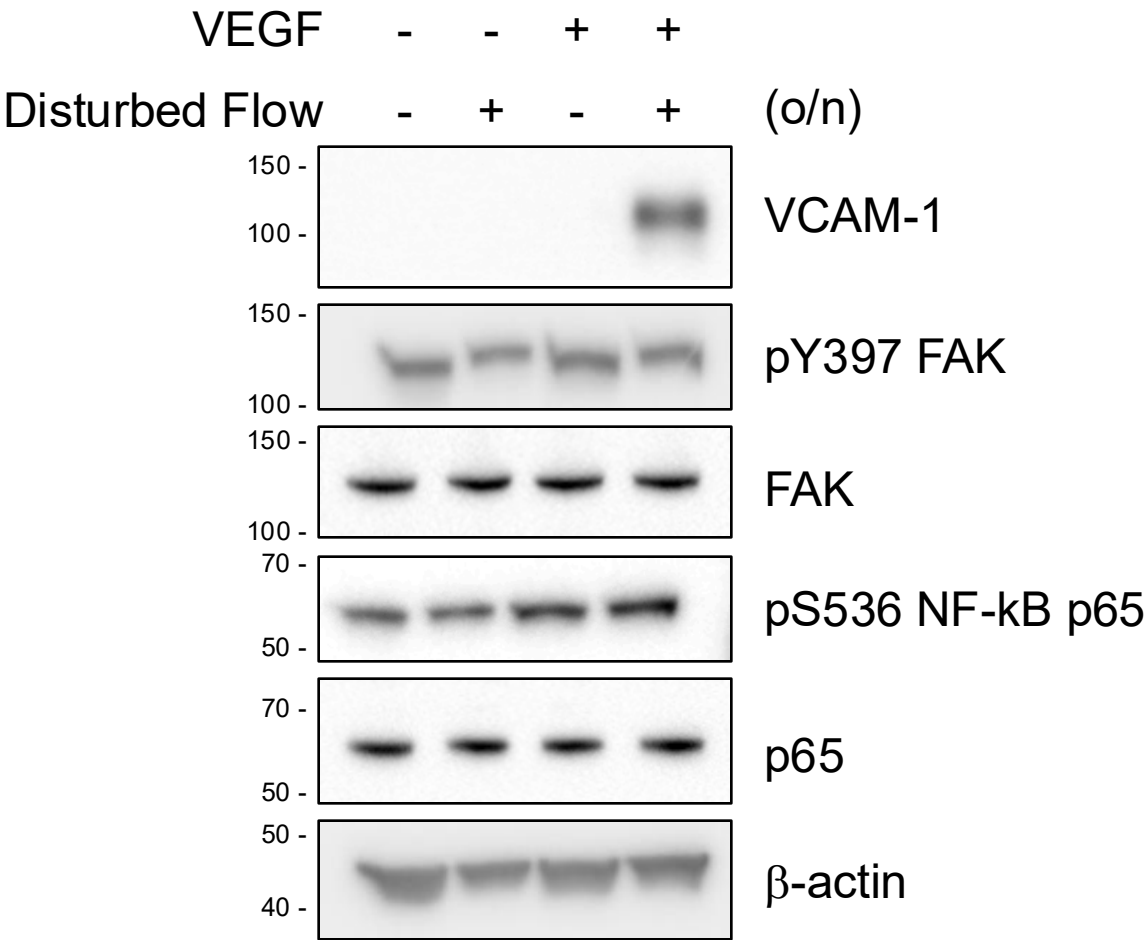

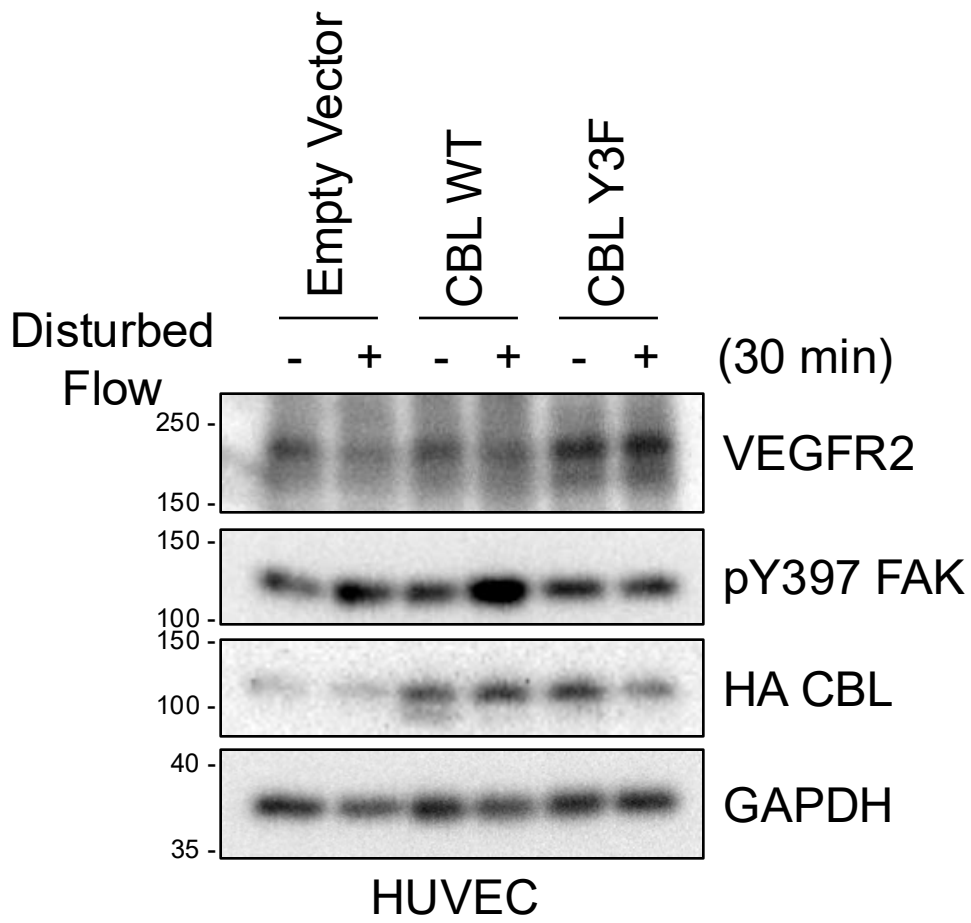

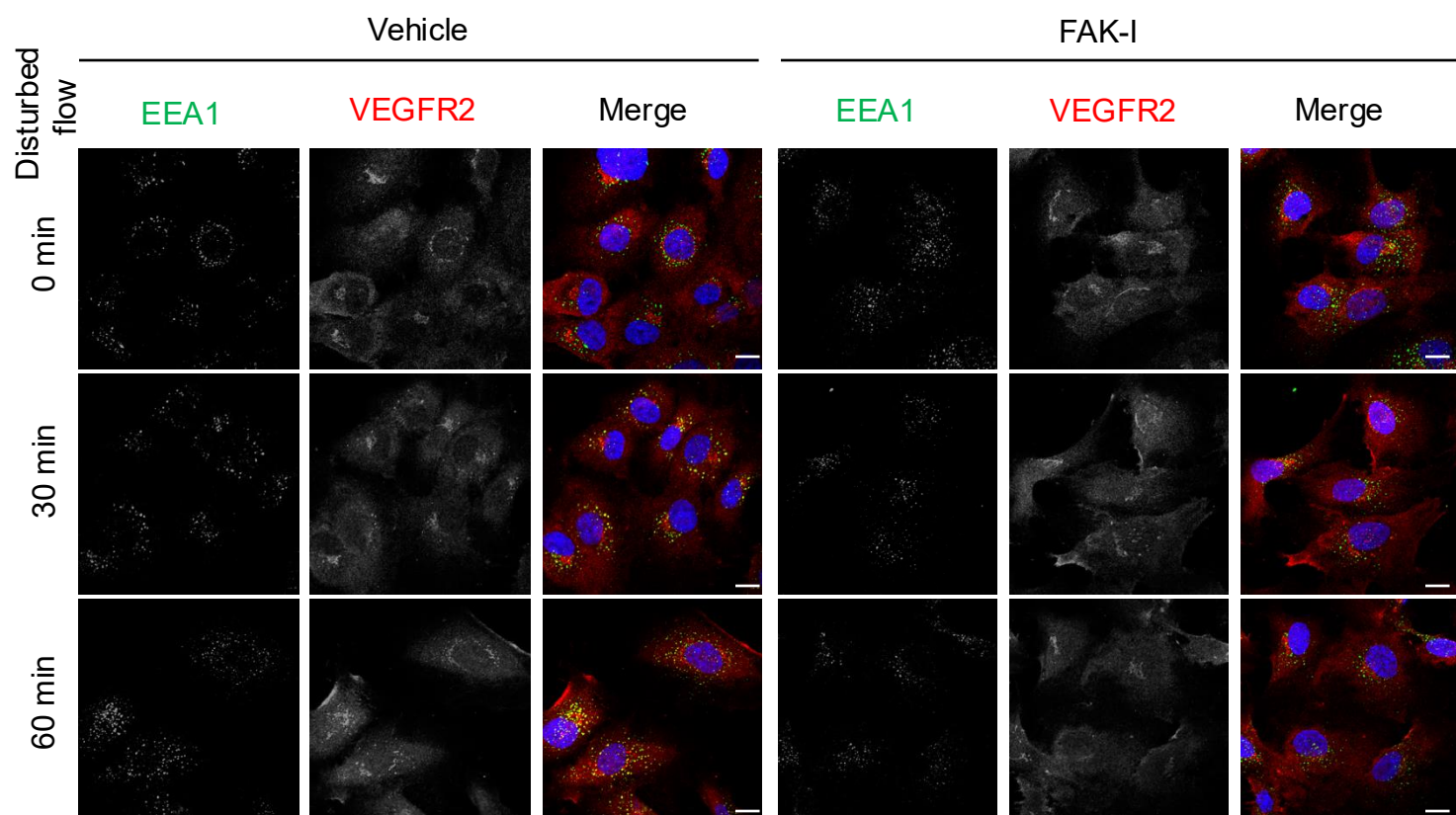

**A**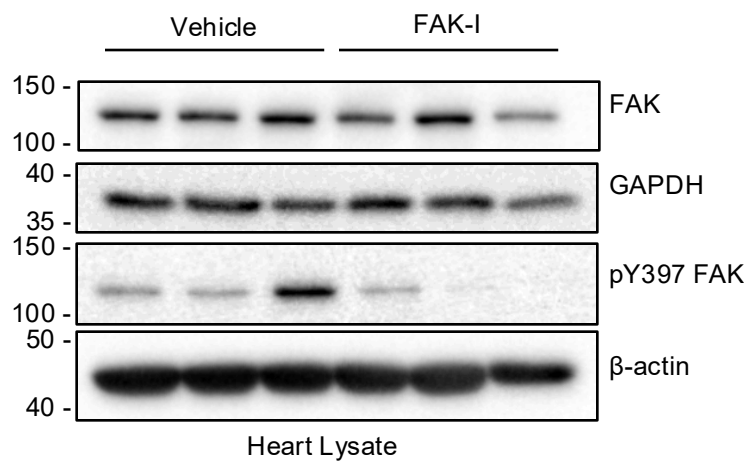**B**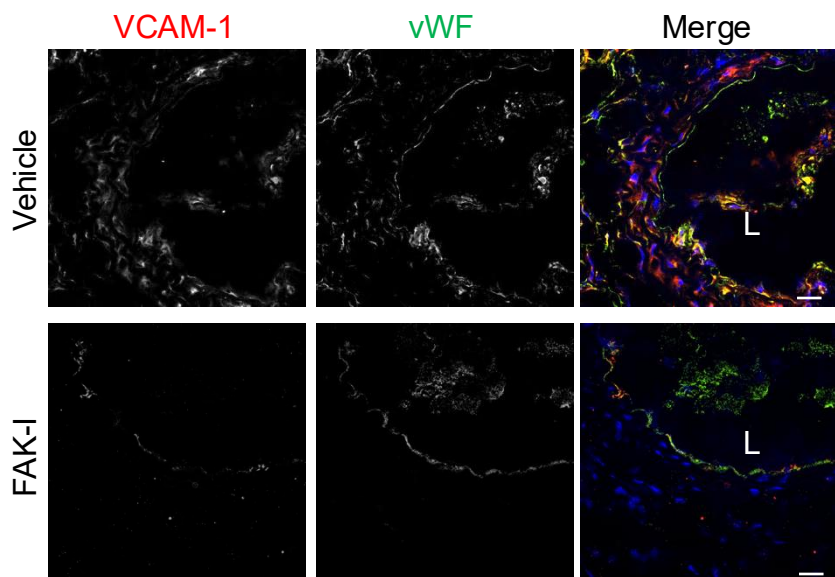**C**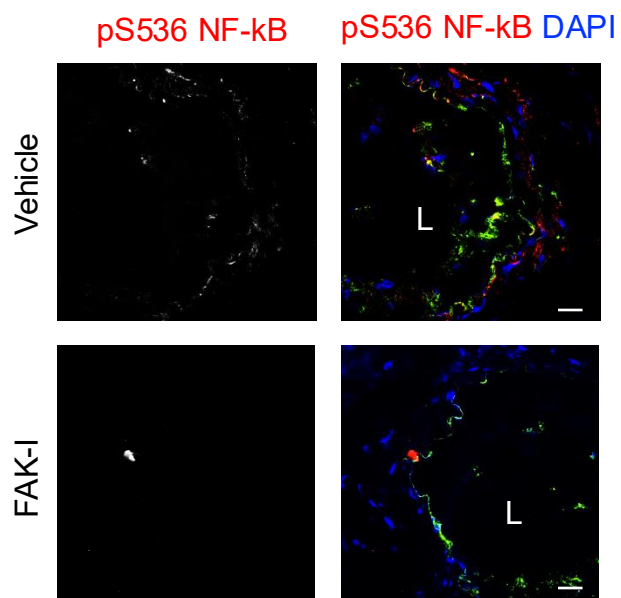**D**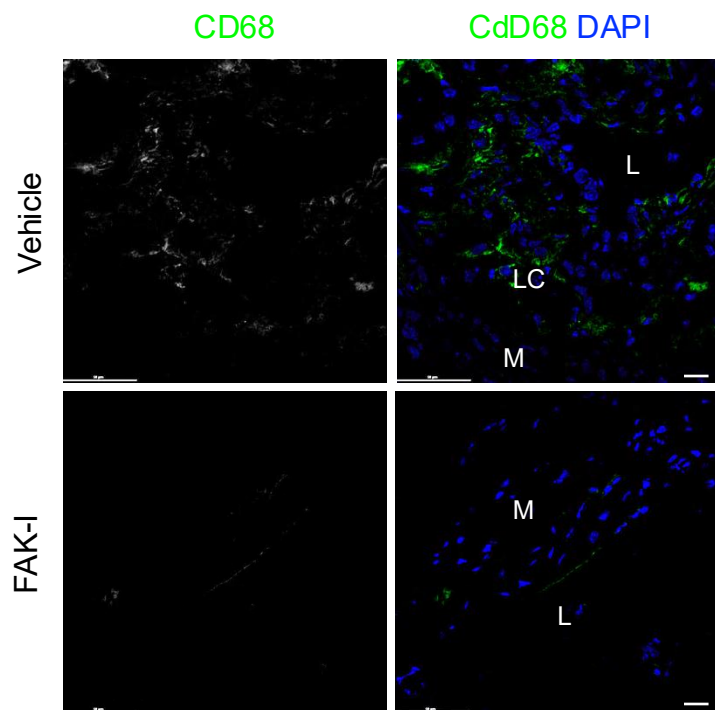

**A**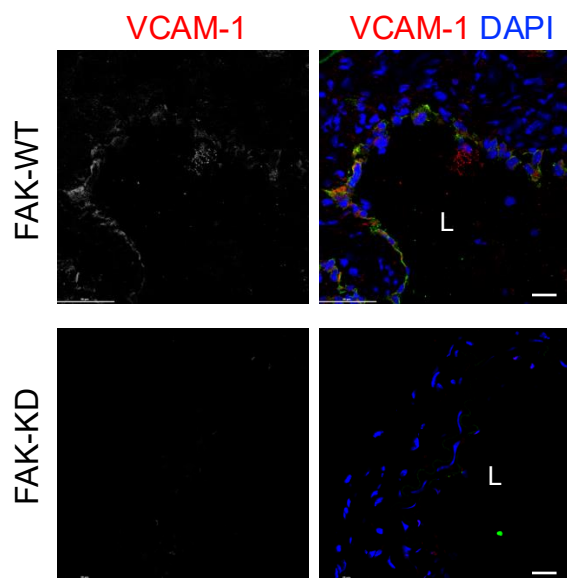**B**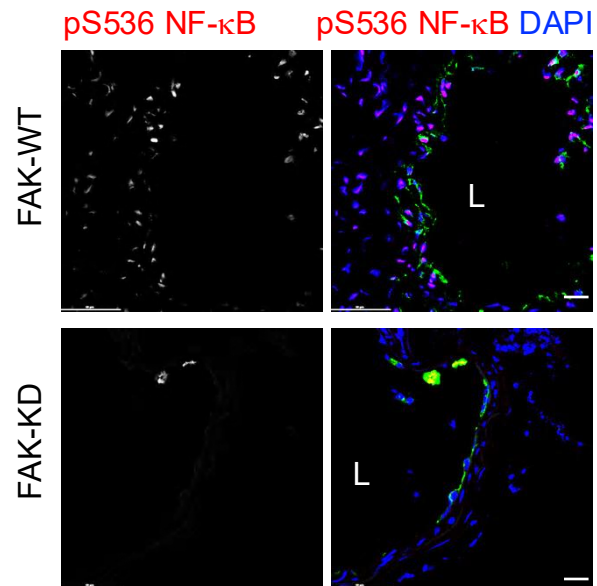

Supplement: Supplementary Figures [file mmc2.pdf]
